# The role of ATP in the differential ability of Sr^2+^ to trigger Ca^2+^ oscillations in mouse and human eggs

**DOI:** 10.1093/molehr/gaaa086

**Published:** 2021-01-05

**Authors:** Anna Storey, Khalil Elgmati, Yisu Wang, Paul Knaggs, Karl Swann

**Affiliations:** 1 Wales Fertility Institute, University Hospital of Wales, Cardiff, UK; 2 School of Medicine, Cardiff University, Cardiff, UK; 3 School of Biosiences, Cardiff University, Cardiff, UK

**Keywords:** oocyte, calcium, intracellular ions, cell signaling, assisted oocyte activation

## Abstract

At fertilization in mice and humans, the activation of the egg is caused by a series of repetitive Ca^2+^ oscillations which are initiated by phospholipase-C(zeta)ζ that generates inositol-1,4,5-trisphophate (InsP_3_). Ca^2+^ oscillations and egg activation can be triggered in mature mouse eggs by incubation in Sr^2+^ containing medium, but this does not appear to be effective in human eggs. Here, we have investigated the reason for this apparent difference using mouse eggs, and human eggs that failed to fertilize after IVF or ICSI. Mouse eggs incubated in Ca^2+^-free, Sr^2+^-containing medium immediately underwent Ca^2+^ oscillations but human eggs consistently failed to undergo Ca^2+^ oscillations in the same Sr^2+^ medium. We tested the InsP_3_-receptor (IP3R) sensitivity directly by photo-release of caged InsP_3_ and found that mouse eggs were about 10 times more sensitive to InsP_3_ than human eggs. There were no major differences in the Ca^2+^ store content between mouse and human eggs. However, we found that the ATP concentration was consistently higher in mouse compared to human eggs. When ATP levels were lowered in mouse eggs by incubation in pyruvate-free medium, Sr^2+^ failed to cause Ca^2+^ oscillations. When pyruvate was added back to these eggs, the ATP levels increased and Ca^2+^ oscillations were induced. This suggests that ATP modulates the ability of Sr^2+^ to stimulate IP3R-induced Ca^2+^ release in eggs. We suggest that human eggs may be unresponsive to Sr^2+^ medium because they have a lower level of cytosolic ATP.

## Introduction

At fertilization in all mammals the sperm activates development of the metaphase II (MII) arrested oocyte (hereafter referred to as an egg) by triggering a prolonged series of transient increases in the intracellular free Ca^2+^ ion concentration (Kline and Kline, 1992; Miyazaki, 2007). These are commonly referred to as Ca^2+^ oscillations and are essential for the completion of meiosis and cortical granule exocytosis (Miyazaki, 2007; Sanders and Swann, 2016) The sperm initiates these Ca^2+^ oscillations after gamete fusion by introducing the sperm specific protein phospholipase c zeta (PLCzeta)(ζ) into the egg cytoplasm where it generates inositol 1,4,5-trisphophate (InsP_3_) (Sanders and Swann, 2016; Swann and Lai, 2016; Wakai *et al.*, 2019). Ca^2+^ oscillations are also seen after ICSI in both mouse and human eggs (Miyazaki, 2007; Hachem *et al.*, 2017; Nozawa *et al.*, 2018; Ferrer-Buitrago *et al.*, 2018b). Notably, PLCζ injection, either as cRNA or as recombinant protein, can trigger prolonged Ca^2+^ oscillations in mouse and human eggs as well as in eggs from other mammalian species (Rogers *et al.*, 2004; Miyazaki, 2007; Swann *et al.*, 2012; Swann and Lai, 2016).

There is a persistent incidence of cases of male factor infertility where failed fertilization occurs after ICSI (Ferrer-Buitrago *et al.*, 2018b). Many of these are due to failed egg (oocyte) activation, which is associated with a deficiency in PLCζ levels, or with specific mutations in PLCζ that lead to loss of its enzyme activity (Escoffier *et al.*, 2016; Ferrer-Buitrago *et al.*, 2018b). These and other cases of failed fertilization can be rescued by artificial egg activation, but the efficiency of these protocols for rescuing fertilization is unclear. The most commonly used activating agents for human eggs are the ionophores A23187 and ionomycin, but they both cause a single large Ca^2+^ increase that fails to mimic the Ca^2+^ oscillations seen at fertilization (Swann, 2018; Ferrer-Buitrago *et al.*, 2018a). Several studies suggest that triggering a single Ca^2+^ increase is less effective at activating development than causing multiple Ca^2+^ increases (Bos-Mikich *et al.*, 1995; Ducibella *et al.*, 2002; Ferrer-Buitrago *et al.*, 2018a). In unfertilized mouse and rat eggs parthenogenetic egg activation is achieved with high success rates and reliability by incubation in (Ca^2+^ free) Sr^2+^ containing medium (Kline and Kline, 1992; Bos-Mikich *et al.*, 1995; Tomashov-Matar *et al.*, 2005). Sr^2+^ medium is more effective in rodent eggs because it causes prolonged Ca^2+^ oscillations that mimic to a considerable extent the oscillations seen at fertilization. Sr^2+^ medium is as effective as PLCζ in activating development of mouse eggs up to the blastocyst stage (Yu *et al.*, 2008; Ferrer-Buitrago *et al.*, 2018a). However, Sr^2+^ medium is not used in cow or pig eggs, despite the simplicity of its use and a clear need for an activation stimulus, because it has never been shown to cause Ca^2+^ oscillations after ICSI in these species (Swann, 2018; Ferrer-Buitrago *et al.*, 2018a). Some studies have suggested that Sr^2+^ medium may be effective in activating human eggs (Yanagida *et al.*, 2006; Fawzy *et al.*, 2018). However, exposing failed-to-fertilize eggs to Sr^2+^ containing medium has not been widely adopted as an activating agent in clinical IVF and it has never been shown that it can cause Ca^2+^ oscillations in human eggs (Rogers *et al.*, 2004; Swann, 2018; Ferrer-Buitrago *et al.*, 2018a). One study has shown that Sr^2+^ does not cause Ca^2+^ oscillations in *in vitro* matured or freshly ovulated unfertilized human eggs (Lu *et al.*, 2018), but the effects of Sr^2+^ on eggs that have failed to fertilize after IVF or ICSI have not been reported. Sr^2+^ enters eggs via the TRPV3 receptor, which is expressed and functional in both mouse and human eggs, so there is no obvious reason why Sr^2+^ should not permeate both mouse and human eggs (Lu *et al.*, 2018; Swann, 2018). The difference between species may be related to a difference in intracellular release.

PLCζ and Sr^2+^ both stimulate release of Ca^2+^ in eggs via the InsP_3_ receptor (IP3R) (Miyazaki, 2007; Wakai *et al.*, 2019). PLCζ generates regenerative cycles of InsP_3_ production that lead to IP3R-induced Ca^2+^ release (Sanders *et al.*, 2018; Matsu-Ura *et al.*, 2019). Sr^2+^ does not appear to generate InsP_3_ because, unlike fertilization and PLCζ, Sr^2+^-induced Ca^2+^ oscillations do not lead to IP3R downregulation (Brind *et al.*, 2000; Jellerette *et al.*, 2000). Instead, it has been shown that Sr^2+^ sensitizes the IP3R to InsP_3_-induced Ca^2+^ release in mouse eggs (Sanders *et al.*, 2018). Ca^2+^ release and oscillations in mouse eggs have been shown to be mediated via the type I IP3R, ITPR1 (Miyazaki, 2007; Wakai *et al.*, 2019). This is the predominant IP3R isoform found in mouse eggs and it is also detected in comparable amounts in mature MII human eggs (Brind *et al.*, 2000; Jellerette *et al.*, 2000; Goud *et al.*, 2002; Mann *et al.*, 2010). IP3Rs are found in clusters of the endoplasmic reticulum in both mouse and human eggs (Mehlmann *et al.*, 1995; Mann *et al.*, 2010). Ca^2+^ transients can be elicited in mouse and human eggs by injection of InsP_3_, or by application of thimerosal, which stimulates IP3Rs (Homa and Swann, 1994; Kline and Kline, 1994; Herbert *et al.*, 1995; Mann *et al.*, 2010). The concentration range of InsP_3_ or thimerosal used to stimulate Ca^2+^ release in mouse and human eggs overlaps. Consequently, whilst a difference in IP3Rs could underlie the species differences in mouse and human egg sensitivity to Sr^2+^, it is not clear whether any difference exists. The IP3R can be regulated by a range of factors such as phosphorylation and Ca^2+^ store content (Galione *et al.*, 1993; Wakai *et al.*, 2013, 2019). IP3Rs also have a specific cytosolic binding site for ATP which can modulate the channel and promote Ca^2+^ release (Foskett *et al.*, 2007). It is not clear if any of these factors might modulate the IP3R sensitivity in mammalian eggs.

In this study, we show that human eggs that had failed to fertilize after IVF or ICSI do not display Ca^2+^ oscillations in response to Sr^2+^ medium that causes immediate Ca^2+^ oscillations in mouse eggs. We show that this lack of sensitivity to Sr^2+^ in human eggs is correlated with an order of magnitude difference in the sensitivity of InsP_3_ induced Ca^2+^ release. We find that a medium that can reduce the level of ATP in mouse eggs makes them unresponsive to Sr^2+^ medium, in a way that is fully reversible. Interestingly, there is a distinctive and consistent difference in the usual concentration of ATP between these two species, with mouse eggs having approximately twice the level of human eggs. This suggests that different levels of ATP could explain the differential sensitivity of the IP3R to Sr^2+^ between mouse and human eggs.

## Materials and methods

### Egg collection and preparation

Mouse eggs were collected from two different strains of mice. MF1 mice were used in early work and the CD1 strain in later studies because of the lack availability of MF1 mice within the UK. The female MF1 mice (6–10 weeks old) were super-ovulated by serial i.p. injections of pregnant mare’s serum gonadotropin and HCG, about 48 h apart. The CD1 female mice (8–12 weeks old) were super-ovulated by serial i.p. injections of PG600, about 48 h apart (all hormones from MSD Animal Health UK Ltd, Milton Keynes, UK). All procedures were carried out under a UK Home Office Project Licence held by KS. Mice were housed in conventional cages with environmental enrichment on a 12 h dark light cycle. For either mouse strain, 15 h after the second hormone injection the mice were culled by cervical dislocation. The oviducts were dissected and then the cumulus-oocyte masses were transferred to M2 medium (Sigma-Aldrich Co Ltd, Gillingham, UK) containing hyaluronidase (Campbell and Swann, 2006; Yu *et al.*, 2008). After the dispersion of cumulus cells by hyaluronidase, the eggs were washed and then maintained in M2 medium at 37°C under mineral oil until fluorescence recordings began. Some mouse eggs were ‘aged’ *in vitro* by holding in M2 medium overnight and they were then used in experiments the next morning, at 24–26 h after egg collection. In mouse egg experiments the ‘n’ numbers refer to the numbers of eggs, but every experiment was from at least two, and usually three, independent days with eggs collected from at least two mice per day.

Human eggs that had failed to fertilize were obtained from patients attending the Wales Fertility Institute for IVF treatment. All patients providing such eggs gave written informed consent to the research. The project was approved by the South East Wales Ethics Committee 2 and is licenced by the Human Fertilisation and Embryology Authority (R0161). Patients donating eggs used in this study had a mean age of 33.59 years (±4.65 SD, n = 51) and mean BMI of 24.27 kg/m^2^ (±3.16 SD). Unfertilized eggs were identified 16–18 h after insemination or sperm injection and transferred from the clinic to the research laboratory in a heated transporter within 3 h, so that experiments were initiated within 24 h of egg recovery. Eggs from each patient were processed on a different experimental day that covered a period of up to 3 years. Only eggs that showed no sign of activation (neither 2nd polar bodies nor a pronucleus) from failed IVF or ICSI were used. Eggs that contained any obvious vacuoles in the cytoplasm were not used for research. The eggs were maintained in the research laboratory in the M2 medium at 37°C under the same conditions as mouse eggs.

### Microinjection of eggs

Mouse or human eggs were microinjected using pressure pulses applied to the back of a micropipette that was inserted into the egg using electrical oscillation, as described in detail elsewhere (FitzHarris *et al.*, 2018). It involves using a sharp tipped micropipette that is back filled with injection medium and inserted into the egg membrane by electrical oscillation on an amplifier connected to the injection solution and bath of medium. A pressure pulse is then applied to the back of the micropipette holder via a tube connected to a pressure pump. Most experiments involved microinjecting Oregon Green BAPTA dextran (Thermo-Fisher, UK) (OGBD: 0.5 mM in the injection pipette in a KCl buffer) (Swann, 2013). In some cases, Cal520 dextran (Stratech Scientific Ltd, Ely, UK) was used as an alternative Ca^2+^ dye that has a similar Ca^2+^ affinity and spectral properties to OGBD. In either case the dextran tag ensures that the dye is retained within the cytosolic compartment of the egg. For experiments on the IP3R sensitivity we microinjected eggs with a mixture of 0.5 mM NPE-Caged InsP_3_ (Thermo-Fisher, UK) plus 0.5 mM OGBD. With both mouse and human eggs, the volume injected was about ∼¼ diameter (∼2% of the egg volume) in order to make the relative amount of caged InsP_3_ and OGBD similar for the differently sized human and mouse egg.

### Medium for experimental runs

Mouse or human eggs were tested for responses to Sr^2+^ in different media. Sr^2+^ containing HKSOM consisted of 95 mM NaCl, 2.5 mM KCl, 0.35 mM KH_2_PO_4_, 0.2 mM MgSO4, 4 mM NaHCO_3_, 0.01 mM EDTA, 0.2 mM Na pyruvate, 10 mM Na lactate, 1 mM glutamine, 0.2 mM glucose, 0.1 mg/l phenol red and 20 mM HEPES at pH 7.4 to which SrCl_2_ was added at the concentrations indicated. In some cases, a Mg^2+^ and Ca^2+^ free M2 was used that consisted of 10 mM SrCl_2_ plus 94.7 mM NaCl, 4.78 mM KCl, 1.19 mM KH_2_PO4, 4 mM NaHCO_3_, 0.33 mM pyruvate, 23.3 mM Na lactate, 5.56 mM glucose, 1 mg/l phenol red and 21 mM Na HEPES at pH 7.4. Other experiments used a HEPES-buffered saline solution (HS) that consisted of 10 mM SrCl_2_ plus 137 mM NaCl, 5.5 mM KCl, 1.2 mM MgCl_2_, 5.6 mM glucose, and 7.5 mM Na HEPES at pH 7.4 (Igusa and Miyazaki, 1983). All media were made up from chemicals and water purchased from Sigma-Aldrich UK Ltd and each was of embryo grade (where available) or else cell culture grade. Serum albumin was omitted so that the eggs adhered to the coverslip of the chamber used for imaging. Reagents, such as thapsigargin or ionomycin (Sigma-Aldrich UK Ltd, Gillingham, UK), were made in stocks of dimethylsulphoxide at 1000 times the working concentrations, and stored at −20°C and diluted to the working concentration on the day of use. In cases where thapsigargin or ionmycin were added to the dish, the zona pellucidas for mouse eggs were removed prior to placement in the recording chamber by brief treatment with acid Tyrode’s solution (Sigma-Aldrich UK Ltd, Ely, UK). In such cases, 100 µl of a solution that was 10X the final concentration was pipetted into the dish of that already contained 900 µl of medium.

### Live imaging of eggs

Eggs were imaged in a 0.9 ml drop of medium that was covered with mineral oil (Sigma-Aldrich UK, Ltd, Ely, UK) in a heated dish (35–37°C) on the stage of an epifluorescence microscope (either a Nikon Eclipse TE2000, Nikon TiU or a Zeiss Axiovert 100). Excitation light was at 490 nm from a halogen lamp or LED (MonoLED, Cairn Research Ltd, Faversham, UK), and emission was at 520–550 nm. The fluorescent light from eggs injected with Ca^2+^-sensitive dyes was sampled and imaged intermittently (every 10 s) with CCD cameras (Photometrics HQ_2_, or Retiga R3), or else we used continuous very low light imaging with an intensified CCD (ICCD) camera (Photek Ltd, St Leonards on Sea, UK). Micromanager Software (https://micro-manager.org/) was used to control the shutters and collect data except for the ICCD, which used specific software (Photek Ltd, St Leonards on Sea, UK). The dyes used are single wavelength indicators and so the fluorescence traces for most experiments have been normalized by plotting the fluorescence as a ratio of each point divided by the starting fluorescence value (hence F/F0), as described previously (Swann, 2013). The time between placing the eggs in the imaging dish and the start of recording was about 1–2 min. When Ca^2+^ oscillations began soon after recording had started, the F0 was taken from the apparent resting level of fluorescence between or at the end of Ca^2+^ oscillations.

For InsP_3_ uncaging experiments, eggs were microinjected under red light and transferred within ∼15 min to a heated microscope stage containing HKSOM medium (Sanders *et al.*, 2018). The eggs were then exposed to pulses of a UV light source that consisted of a metal Halide lamp with a fiber optic guide used to illuminate the dish containing the eggs. The metal Halide light path was filtered by a Schott UG11 filter to select UV light (Thorlabs Ltd, Ely, UK) and this was passed via computer-controlled shutters that enabled UV pulses to be delivered with durations from 100 ms to 10 s. To enable rapid and continuous imaging of Ca^2+^ in these experiments, the OGBD fluorescent light was measured in single eggs continuously with a photomultiplier tube (ET Enterprises Ltd, Uxbridge, UK) with a current to voltage convertor sampled at 10 Hz by an AD convertor connected to a computer. During application of the UV pulse there was a large flux of light that caused an artifactual signal from the photomultiplier tube and so this part of the recording was removed from the traces.

### ATP assays

Whole cell calibrated ATP measurements were performed by adding single eggs to individual tubes containing 200 µl luciferase Promega Glo reagent (Promega Ltd, Southampton, UK). The light signals were taken as the steady state values. Light was recorded using a custom made luminometer consisting of a cooled photomultiplier tube in photon counting mode (ET Enterprises Ltd, Uxbridge, UK). Signals from single eggs were typically 1000 times the background count. The signals were calibrated with a series of dilutions of ATP. In other experiments on live eggs the dynamic changes in (relative) cytosolic ATP levels were measured at the same time as cytosolic Ca^2+^ by injecting firefly luciferase along with OGBD, as described previously (Campbell and Swann, 2006). Luminescence signals from eggs were monitored with a Photek ICCD camera which was configured to switch between luminescence and fluorescence imaging modes every 10 s, as described previously (Campbell and Swann, 2006).

### Statistical analysis

Data sets were analyzed using ImageJ (https://imagej.nih.gov/ij/index.html) and SigmaPlot software (Systat Software Inc, Slough, UK). The error bars used in all cases are SDs. The ‘n’ numbers refer to the number of eggs and were compiled from at least two experimental runs for mouse eggs and involved as many experimental runs as patients’ donations for human eggs. Statistical tests used and *P*-value inequalities refer to Student ‘*t*’ tests when the data passed the Shapiro–Wilk normality test, and the Mann–Whitney rank sum test when data failed the Shapiro–Wilk test. A *P*-value of less than 0.05 was considered to be significant. The effect size (Cohen’s *d*) for two sets of data was calculated by dividing the differences in the means by the averaged SD.

## Results

### Ca^2+^ in mouse and human eggs in response Sr^2+^ medium

**Figure 1.**
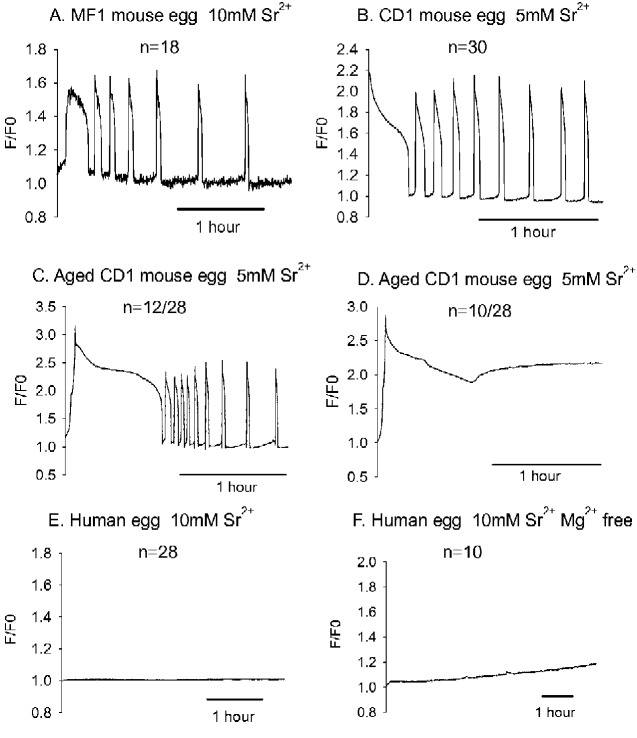
**Ca^2+^ measured in mouse or human eggs incubated in Sr^2+^ containing medium.** (**A**) An example of Ca^2+^ oscillations (measured by Oregon Green BAPTA dextran (OGBD) fluorescence) in a MF1 mouse egg placed in 10 mM Sr^2+^ medium, where 7.1 ± 2.5 (mean ± SD) Ca^2+^ transients were seen in 18/20 eggs in 2 h, with two other eggs showing a sustained Ca^2+^ increase followed by lysis. (**B**) An example of Ca^2+^ oscillations in a CD1 mouse egg in response to 5 mM Sr^2+^ medium, where 5.6 ± 3.5 spikes were seen in 30 eggs in 2 h with another six eggs showing a sustained rise in Ca^2+^ and lysis. In (**C**) is shown an example of 12/28 mouse eggs (CD1) that had been aged in vitro (∼24 h) that also underwent Ca^2+^ oscillations in 5 mM Sr^2+^ containing medium. In (**D**) is shown another example of one of 10/28 aged mouse eggs that showed a sustained Ca^2+^ increase in 5 mM Sr^2+^ medium during 2 h of recording. In (**E**) an OGBD trace is shown for a human egg placed in medium containing 10 mM Sr^2+^ (one of 28 eggs). (**F**) A trace is shown where Cal520 dextran was used to measure Ca^2+^ after the egg that was placed 10 mM Sr^2+^ with no added Mg^2+^ (one of 10 eggs). F/F0: fluorescence presented as a ratio of each point divided by the starting fluorescence value.

In previous studies, 10 mM Sr^2+^ in Ca^2+^ free (HKSOM) medium was shown to cause Ca^2+^ oscillations and activate MF1 mouse eggs (Yu *et al.*, 2008). Figure 1A shows that typical Ca^2+^ oscillations were detected with OGBD in nearly all MF1 mouse eggs. Mouse eggs from CD1 also underwent sustained intracellular Ca^2+^ oscillations when placed in Ca^2+^ free medium (HKSOM) containing Sr^2+^ (Fig. 1B). With CD1 mouse eggs, medium containing 10 mM Sr^2+^ caused Ca^2+^ oscillations in 4/10 eggs but most eggs underwent a sustained Ca^2+^ increase, which led to cell lysis within 2 h. Consequently, with CD1 mouse eggs, we used medium containing 5 mM Sr^2+^ and all 30 eggs underwent Ca^2+^ oscillations, and only 4 eggs lysed during the recording period. With either strain of mouse egg, the first Ca^2+^ transients typically started either as soon recording was initiated, or within ∼10 min of the start of recording. The initial Ca^2+^ increase was usually long lasting compared to other transients and, in some cases, Ca^2+^ was at a high level for 1 h before oscillations started. All eggs that oscillated within 2 h showed signs of activation in forming second polar bodies. Mouse eggs (CD1) that were aged *in vitro* (∼24 h) also showed Ca^2+^ increases in 5 mM Sr^2+^ containing medium (Fig. 1C and D). However, in aged eggs the Ca^2+^ increase was more variable. A large proportion of eggs (12/28) showed a very prolonged Ca^2+^ increase before oscillating (Fig. 1C), whilst others underwent a sustained Ca^2+^ increase (Fig. 1D) and in two cases egg lysis occurred during the recording. In a further six eggs (not shown) there were Ca^2+^ oscillations before a sustained Ca^2+^ rise. Eggs that underwent a sustained Ca^2+^ rise showed no signs of activation and those that oscillated displayed a fragmented appearance, which is consistent with previous studies on aged mouse oocytes (Gordo *et al.*, 2002). These data show that there are some qualitative differences in the response to Sr^2+^ medium of mouse eggs from different strains and postovulatory ages. However, Ca^2+^ increases and oscillations are rapidly and consistently induced by 5 or 10 mM in HKSOM medium. The only mouse eggs that failed to undergo oscillation with Sr^2+^ medium showed a sustained rise in Ca^2+^ followed by lysis within the 2 h of recording.

When human eggs that had failed to fertilize were placed in 10 mM Sr^2+^ HKSOM medium none of 28 eggs (from eight different patients) underwent Ca^2+^ oscillations for at least 3 h (Fig. 1E). When six of these human eggs (from two patients) were kept in Sr^2+^ medium for over 10 h they also failed to display any Ca^2+^ oscillations. Most of these eggs were from failed ICSI treatments (24/28). We also tested the effect of using a 10 mM Sr^2+^ medium that is both devoid of added Ca^2+^ or Mg^2+^ ions on eggs (Fig. 1F). This medium caused a sustained Ca^2+^ increase and cell lysis in CD-1 mouse eggs (data not shown). However, when this medium was used for human eggs, we failed to observe any Ca^2+^ distinctive oscillations in 10 human eggs in up to 10 h of recording. Out of 10 such eggs, eight showed no oscillations, but many eggs displayed a gradual rise in Ca^2+^ levels (e.g. Fig. 1F). One egg showed a single Ca^2+^ spike after ∼11 h (Supplementary Fig. S1), but this egg failed to show signs of activation, and another showed some irregular Ca^2+^ oscillations followed by lysis. These data show that Sr^2+^ medium, that causes a sustained Ca^2+^ increase in mouse eggs, fails to cause any response in most human eggs within a timeframe that is useful for egg activation. Overall, the data suggest the *in vitro* aged human eggs are unable to undergo regular Ca^2+^ oscillations in response to the same Sr^2+^ medium that causes all mouse eggs to display a Ca^2+^ increase within minutes of exposure.

### InsP_3_ induced Ca^2+^ release in mouse and human eggs

**Figure 2.**
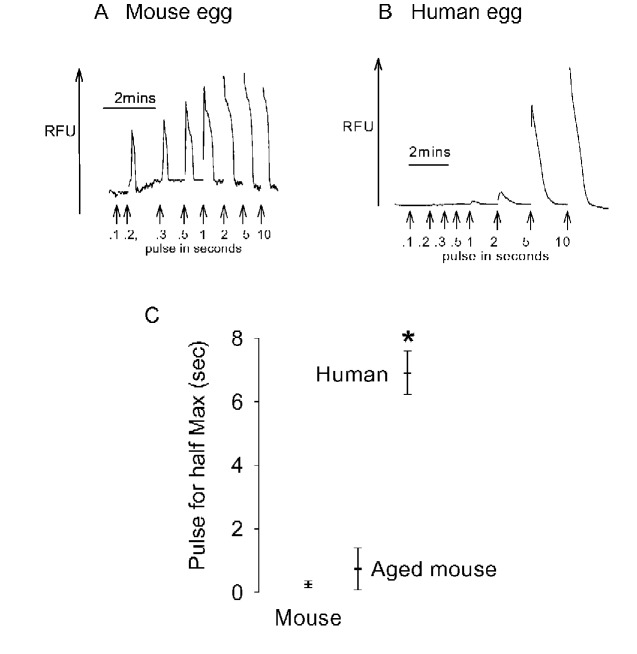
**InsP_3_ induced Ca^2+^ release in CD1 mouse and human eggs using caged InsP_3_.** (**A**) A typical recording where pulses of UV light of 100 ms (0.1 s) to 10 s were applied to a CD1 mouse egg injected with OGBD and caged 1,4,5-trisphophate (InsP_3_). Ca^2+^ increases are seen with all pulse durations over 200 ms. (**B**) A similar experiment except with a human egg. In this case, Ca^2+^ increases were only seen with UV pulses of greater than 2 s. We plotted the amplitude of each Ca^2+^ response against UV pulse duration to estimate the pulse duration that would have given a half maximal increase. The mean (horizontal line) and SD for the half maximal response is shown in (**C**) for human eggs (n = 14), fresh CD1 mouse eggs (n = 13) and aged CD1 mouse eggs (n = 8). (Note that the SD for fresh mouse eggs is small.) The differences between human eggs (*)compared to either fresh or aged mouse eggs are significantly different with *P* < 0.001. RFU, relative fluorescence units.

The lack of response to Sr^2+^ medium in human eggs could be related to differences in the sensitivity of the IP3R. We tested the sensitivity of mouse eggs to InsP_3_ induced Ca^2+^ release by using UV light pulses to uncage InsP_3_ in the cytosol (Sanders *et al.*, 2018). Figure 2A shows Ca^2+^ transients were triggered by pulses of UV light in caged InsP_3_ injected CD1 mouse eggs. A pulse duration of around 200 ms consistently induced a small and transient Ca^2+^ increase, whereas pulse durations of 1–2 s induced a maximal Ca^2+^ increase responses (Fig. 2A). In contrast, the same protocol failed to induce a Ca^2+^ increase in human eggs when pulses of up to 2 s were applied (Fig. 2B). In human eggs, UV pulses of around 5–10 s induced a large Ca^2+^ increase. Figure 2 shows the size of each OGBD fluorescence increase against the duration of the UV pulse. We used the amplitudes of the fluorescence increases to estimate the theoretical duration of UV pulse required to cause a half-maximal response for each egg. Figure 2C shows that the half maximal response for human eggs was about 10 times greater than that for CD1 mouse eggs, with a clear separation of all data points (an effect size of 13, *P*-value < 0.001). This difference between mouse and human eggs was not likely due to the *in vitro* ageing of human eggs because when we tested CD1 mouse eggs that had been aged *in vitro* for 24 h, we found that they also responded to UV pulses of much shorter duration than that of human eggs (Fig. 2C, *P*-value < 0.001). These data show that there is a marked difference in the sensitivity of mouse and human eggs to InsP_3_ induced Ca^2+^ release.

### Ca^2+^ stores in mouse and human eggs

**Figure 3.**
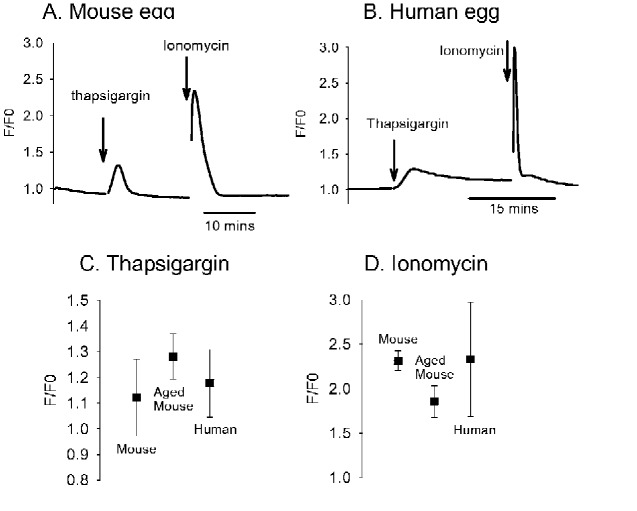
**Ca^2+^ release from eggs in response to thapsigargin and ionomycin.** The data shown are from CD1 mouse and human eggs in Ca^2+^ free HKSOM medium (with 1 mM EGTA added). (**A**) An example of one of 10 fresh mouse eggs responding to addition of 10 µM thapsigargin and then to 5 µM ionomycin and (**B**) is a similar experiment with one of eight human eggs. (**C**) and (**D**) The mean and SDs of the amplitudes of Ca^2+^ transients in response to thapsigargin and then ionomycin in 10 mouse eggs, eight aged mouse eggs and eight human eggs. The difference in response amplitudes were not statistically significant (*P* > 0.05).

The differences in the response of mouse and human eggs to both Sr^2+^ and InsP_3_ induced Ca^2+^ release implies that there is some factor differentially modulating IP3Rs. One possibility is a difference in the amount of Ca^2+^ stored in the egg endoplasmic reticulum between species. As above we injected eggs with OGBD and measured the Ca^2+^ release response to the additions of thapsigargin and ionomycin in Ca^2+^ free medium containing EGTA. Thapsigargin inhibits Ca^2+^ pumps and releases Ca^2+^ from the endoplasmic reticulum and then ionomycin releases Ca^2+^ from all stores. Figure 3A and B shows Ca^2+^ increases in CD1 mouse and human eggs, respectively, in response to thapsigagin and ionomycin. The responses to thapsigargin were smaller than those seen in response to ionomycin in both CD1 mouse and human eggs (Fig. 3C and D). However, with both thapsigargin and ionomycin, there was no significant difference in the amplitude of the Ca^2+^ increases between CD1 mouse (fresh or aged) and human eggs (all *P*-values > 0.05). These data suggest that human eggs have a more variable Ca^2+^ store content than mouse eggs. However, there is a considerable overlap in the responses between mouse and human eggs, which makes it unlikely that differences in Ca^2+^ store content can provide an explanation for marked differences in response to Sr^2+^, and in IP3R sensitivity.

### The effects of pyruvate deprivation on Sr^2+^ induced Ca^2+^ oscillations

**Figure 4.**
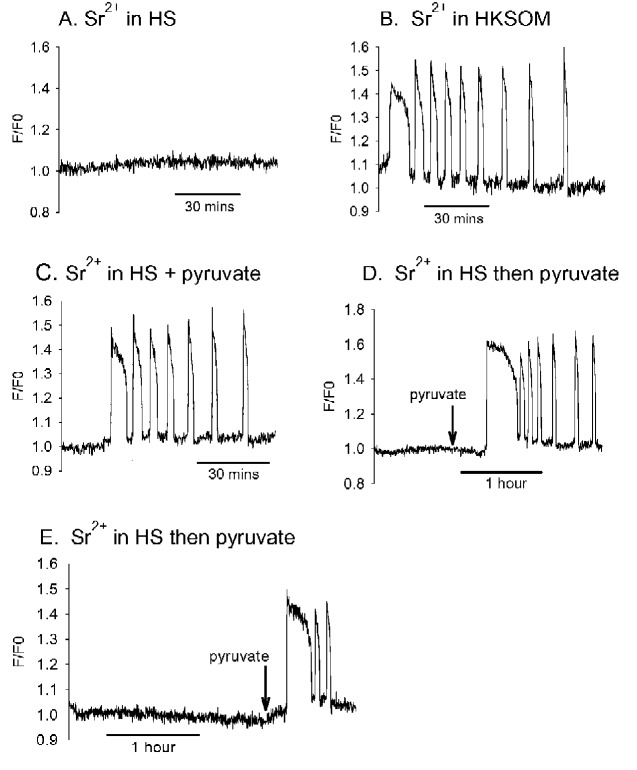
**Ca^2+^ responses in MF1 mouse eggs in 10 mM Sr^2+^ medium.** (**A**) One of 32 mouse eggs that all failed to respond to 10 mM Sr^2+^ in HEPES-buffered saline (HS) medium. (**B**) One of 25/25 eggs (on the same day) that responded to 10 mM Sr^2+^ medium in HKSOM and showed 7.04 ± 2.98 Ca^2+^ spikes in 2 h. (**C**) One of 11/14 mouse eggs that showed Ca^2+^ oscillations in 10 mM Sr^2+^ HS medium containing 0.2 mM pyruvate from the start. In this case, a mean of 5.64 ± 2.77 Ca^2+^ spikes were recorded in 90 min and three other eggs showed a Ca^2+^ rise that never recovered. (**D**) An example of a mouse egg in 10 mM Sr^2+^ in HS where pyruvate was added to the dish later to a final concentration of 0.2 mM. In (**D**), the pyruvate was added after ∼1 h and 14/16 eggs responded by showing Ca^2+^ oscillations, with a mean of 5.78 ± 1.63 spikes in 2 h. In (**E**), pyruvate was added after ∼2 h and Ca^2+^ oscillations where induced in 20/32 eggs, with a mean of 3.05 ± 1.23 spikes in 1 h, with four eggs failing to respond, three lysing and five generating a sustained rise in Ca^2+^.

While carrying out experiments on the effects of divalent cations, we found that one type of Sr^2+^ medium failed to trigger Ca^2+^ oscillations in mouse eggs. A HEPES-buffered saline medium has previously been used to study the mechanism of Ca^2+^ influx in hamster eggs (Igusa and Miyazaki, 1983). It consists of similar salts to HKSOM and M2 medium and contains glucose, but no pyruvate or lactate. When MF1 mouse eggs were placed in this HS medium containing 10 mM Sr^2+^ (with no added Ca^2+^) none of 32 eggs showed any Ca^2+^ oscillations or signs of activation (Fig. 4A). This contrasted with Ca^2+^ oscillations that were seen in all 25 eggs tested on the same experimental days using 10 mM Sr^2+^ HKSOM medium (Fig. 4B). We have previously shown that pyruvate deprivation leads to a decrease in ATP levels, that is fully reversed by adding pyruvate (but not lactate) back to the medium (Dumollard *et al.*, 2004, 2008). We added 0.2 mM pyruvate to the Sr^2+^ containing HS, before the start of recordings, and found that nearly all eggs (11/14) underwent Ca^2+^ oscillations and activated (Fig. 4C). This suggests that pyruvate alone is sufficient to restore sensitivity to Sr^2+^. We then incubated mouse eggs in Sr^2+^ HS (with no pyruvate) from the start of the recording (Fig. 4D and E). As in Fig. 4A, there no Ca^2+^ oscillations, but when we added pyruvate back to the medium after 1 or after 2 h of recording, we found that the addition of pyruvate causes most eggs to undergo Ca^2+^ oscillations. These data show that pyruvate is essential for Sr^2+^ induced Ca^2+^ oscillations in MF1 mouse eggs. This suggests that ATP levels could play a role in sensitizing mouse eggs to Sr^2+^ medium.

**Figure 5.**
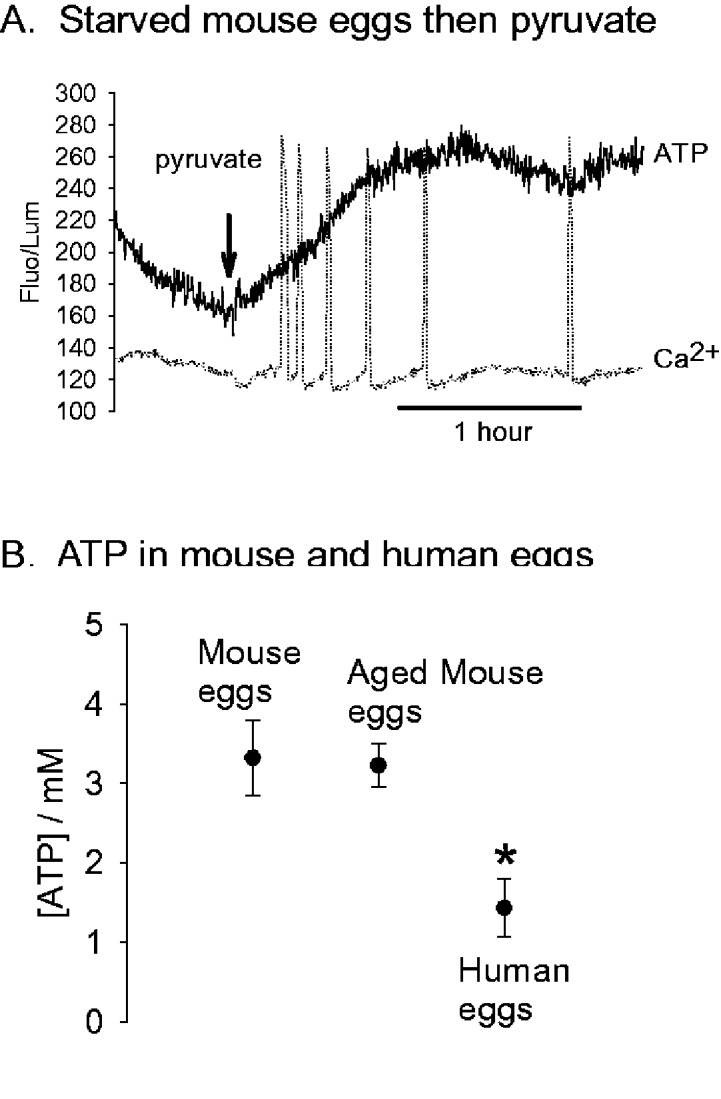
**ATP concentrations in mouse and human eggs.** (**A**) A recording of Ca^2+^ and ATP (using firefly luciferase) in CD1 mouse eggs in HKSOM medium devoid of metabolites (hence eggs were starved). ATP is represented by the solid line and Ca^2+^ by the dotted line. In a total of 18 eggs, there were no Ca^2+^ oscillations in the initial recording period, but after adding back 1 mM pyruvate after 30–40 min all 18 eggs underwent Ca^2+^ oscillations with a frequency of 4.5 Ca^2+^ (±1.4) spikes in 1 h after adding pyruvate. (**B**) The ATP levels in mouse versus human eggs and illustrates the difference (mean and SD) between CD1 mouse eggs (n = 37), aged CD mouse eggs (n = 34) and human eggs (n = 38). The difference in ATP between human eggs (*) and mouse eggs (fresh or aged) is statistically significant (*P* < 0.001).

We repeated these experiments with medium deficient in pyruvate on CD1 mouse eggs, but also measured the dynamics of ATP changes by monitoring the luminescence of firefly luciferase, which indicates ATP levels (Campbell and Swann, 2006; Dumollard *et al.*, 2008). In initial studies we found that some CD1 mouse eggs underwent a Ca^2+^ increase in response to pyruvate-free 5 mM Sr^2+^ medium and when this happened the Ca^2+^ level remained elevated (Dumollard *et al.*, 2008). Consequently, we pre-incubated CD1 mouse eggs in HKSOM without the substrates pyruvate, glucose, glutamine or lactate (but with Ca^2+^) for 30 min before adding them to the same substrate-free HKSOM containing 5 mM Sr^2+^. Figure 5A shows an example of recordings of Ca^2+^ levels in CD1 mouse eggs in substrate-free medium containing 5 mM Sr^2+^, with the luciferase trace indicating the relative level of ATP. There were no Ca^2+^ oscillations in any of the 18 eggs tested and the ATP level continued to decline. However, when pyruvate was added to the medium there was rapid increase in ATP levels and a series of Ca^2+^ oscillations was observed in all eggs. These data support experiments on MF1 mouse eggs and show that a reduction in ATP leads to a reversible loss of sensitivity to Sr^2+^ induced Ca^2+^ oscillations. This implies that the ATP level plays a significant role in determining whether an egg undergoes Ca^2+^ oscillations in response to Sr^2+^ medium.

### Differences in ATP levels between mouse and human eggs

We measured the concentration of ATP in whole individual CD1 mouse eggs and human eggs using a standard luciferase luminescence assay. The concentration of ATP was estimated after taking into account the different sizes of human eggs. Figure 5B shows the distribution of values obtained from different mouse and human eggs. The ATP level in CD1 mouse eggs was 3.32 ± 0.47 mM and in human eggs 1.43 ± 0.37 mM (mean ± SD). There was a marked difference in the estimated ATP levels, with virtually no overlap in the total range of data and a separation of the mean values by more than 4 SDs (an effect size of 4.5, *P*-value < 0.001). There was no significant decline in ATP during *in vitro* ageing of mouse eggs, and mouse eggs aged for ∼24 h post collection still had a higher ATP than that of human eggs (Fig. 5B, *P* < 0.001). These data suggest that the ATP content is significantly lower in human eggs compared to mouse eggs.

## Discussion

We have found that human eggs that have failed to fertilize after IVF or ICSI (and hence they were aged *in vitro*) do not display Ca^2+^ oscillations or activate in response to Sr^2+^ medium that is otherwise effective at inducing immediate Ca^2+^ oscillations in mouse eggs. Mammalian eggs that are aged *in vitro* show a number of biochemical changes that are not seen in freshly ovulated eggs (Szpila *et al.*, 2019). However, we found that *in vitro* aged mouse eggs reliably show Ca^2+^ increases in response to Sr^2+^ medium and so a lack of response in human eggs is unlikely to be caused by *in vitro* ageing itself. Moreover, we have previously found that *in vitro* aged human eggs from failed ICSI or IVF can undergo sustained Ca^2+^ oscillations in response to PLCζ injection (Rogers *et al.*, 2004; Swann *et al.*, 2012). In addition, our data are consistent with a previous report that found Sr^2+^ was unable to trigger Ca^2+^ oscillations in fresh or unfertilized human eggs (Lu *et al.*, 2018). In our study, one human egg showed a single Ca^2+^ spike after more than 10 h in 10 mM Sr^2+^, showing that human eggs are capable of occasionally generating a Ca^2+^ spike in Sr^2+^ medium. However, even this response to Sr^2+^ was after >10 h and the human eggs did not activate. It seems unlikely that Sr^2+^ would have caused Ca^2+^ oscillations in any of the previous clinical reports where it has been claimed to have been effective in egg activation (Yanagida *et al.*, 2006; Fawzy *et al.*, 2018), not least because these studies were based upon incubating human eggs in 10 mM Sr^2+^ medium for 30 min or 1 h, which is within a timeframe that we have never seen any Ca^2+^ increases. A previous study of Ca^2+^ in human eggs suggested that the problem with Sr^2+^ medium is not a lack of Sr^2+^ influx into the egg because human eggs possess the same TRPV3 channels that mediate Sr^2+^ influx (Lu *et al.*, 2018). Hence the fundamental issue with Sr^2+^ appears to be that the IP3R induced Ca^2+^ release in human eggs is substantially less sensitive to stimulation than that in mouse eggs.

It is known that Sr^2+^ induced Ca^2+^ oscillations in mouse eggs require IP3Rs. The levels of IP3Rs could be lower in human eggs that have failed to fertilize after ICSI since a sperm will have been introduced into the cytoplasm. Downregulation of IP3Rs can occur after fertilization in mammalian eggs (Brind *et al.*, 2000; Jellerette *et al.*, 2000; Lee *et al.*, 2010), but this only occurs in response to PLCζ-induced Ca^2+^ oscillations, which seems unlikely in human eggs that have failed to fertilize. In addition, the level of IP3Rs does not appear to be critical because Sr^2+^ can cause Ca^2+^ oscillations in fertilized eggs (zygotes) that have downregulated the level of IP3Rs by several fold in response to InsP_3_ production (Zhang *et al.*, 2005). Sr^2+^ induced oscillations are associated with a marked increase in the sensitivity of mouse eggs to InsP_3_ (Sanders *et al.*, 2018), which is consistent with studies of the type 1 InsP_3_ receptor (ITPR1) cerebellar microsomes where Sr^2+^ directly promotes InsP_3_ induced Ca^2+^ release (Hannaert-Merah *et al.*, 1995). Hence, Sr^2+^ stimulates the ITPR1 and we would expect that the difference between mouse and human eggs should be due to differences in its regulation. Previous studies have shown that InsP_3_ microinjection causes Ca^2+^ release in mouse and human eggs at around 100 nM (Kline and Kline, 1994; Mann *et al.*, 2010). Our data, using UV pulses to uncage InsP_3_, show a very large difference in the threshold for initiating InsP_3_ induced Ca^2+^ release between mouse and human eggs. This difference provides a basis for understanding why Sr^2+^ is only able to trigger Ca^2+^ release in mouse eggs. It may also help explain why human PLCζ needs to be about 30 times more potent than mouse PLCζ in causing Ca^2+^ oscillations in eggs (Yu *et al.*, 2008; Swann and Lai, 2016).

Studies using frogs eggs have shown that increasing the Ca^2+^ store content can sensitize IP3R induced Ca^2+^ release (Galione *et al.*, 1993; Yamasaki-Mann and Parker, 2011). It is unclear whether Ca^2+^ store loading affects IP3R sensitivity in mouse eggs (Wakai *et al.*, 2013). We assayed Ca^2+^ release in mouse and human eggs under the same conditions in response to ionomycin, and thapsigargin and ionomycin in Ca^2+^ free medium. The data suggest that the amount of Ca^2+^ releasable by thapsigargin and ionomycin is similar in mouse and human eggs.

We discovered that a medium that was devoid of pyruvate was unable to support Sr^2+^ induced Ca^2+^ oscillations in mouse eggs. Since pyruvate-free medium leads to a reduction in ATP levels (Dumollard *et al.*, 2004, 2008) and ATP is an allosteric modulator of IP3Rs (Foskett *et al.*, 2007), we investigated whether ATP could begin to explain the difference in IP3R sensitivity. We found a clear difference in ATP levels between mouse and human eggs with an approximate 2-fold higher level in mouse compared to humans. There was little overlap in the total range of values; hence a large effect size. There are many reports of the ATP content in mammalian eggs, but since different laboratories have used different protocols and assay kits to measure ATP, it is difficult to make comparison of absolute concentrations across the literature. Most reports cite ATP in pmol/egg rather than as a concentration so any differences may not have been noticed. However, if we take diameters of 72 µm for mouse and 120 µm for human eggs (Griffin *et al.*, 2006), then previous data from the same laboratory suggest values of 3.5 mM for mouse eggs and 1.9 mM for human eggs (Van Blerkom *et al.*, 1995, 2003). If we recalculate data from another study using these diameters then it suggests ATP concentrations of 4.5 mM and 2.2 mM for the mouse versus human egg, respectively (Chi *et al.*, 1988). Hence, our data are consistent with previous studies. It is not obvious why the level of ATP is lower in human eggs compared to mouse eggs. A 2-fold lower cytosolic ATP concentration will have little effect on the free energy of hydrolysis since this depends upon the logarithm of the ratio of ATP to ADP and phosphate concentrations. Moreover, the effect of ATP upon the IP3R is allosteric and does not involve ATP hydrolysis (Foskett *et al.*, 2007). Hence, Ca^2+^ release can potentially be modulated without affecting the ability to pump Ca^2+^ into stores.

It was notable that we found that *in vitro* aged mouse eggs still responded to Sr^2+^ by showing oscillations shortly after placement in Sr^2+^ containing medium. The levels of ATP were not significantly altered in aged mouse oocytes, which supports our hypothesis that high ATP levels confer sensitivity to Sr^2+^. However, we did find that a significant proportion of aged mouse eggs failed to recover from the initial Sr^2+^ induced Ca^2+^ transients and they usually lysed before the end of the experiment. This is consistent with previous reports that mitochondrial ATP production fails to increase in response to Ca^2+^ oscillations in aged mouse eggs (Szpila *et al.*, 2019). Even aged mouse eggs that underwent Sr^2+^ induced Ca^2+^ oscillations failed to activate and instead showed a fragmented appearance. This is again consistent with previous reports showing that apoptosis is triggered in *in vitro* aged mouse eggs in response to sperm factor injection (Gordo *et al.*, 2002). The *in vitro* aged human eggs we used may have a range of defects, such as increased reactive oxygen species and DNA damage, and that could also make them susceptible to lysis or apoptosis. However, loss of Ca^2+^ homeostasis and apoptosis may be less of an issue with human eggs than mouse eggs that are aged *in vitro* because, as noted above, ‘failed to fertilize’ human eggs can undergo prolonged Ca^2+^ oscillations in response to PLCζ and this can trigger development up to the blastocyst stage (Rogers *et al.*, 2004). It is noteworthy that the mouse eggs we use are from mice that were relatively young compared with the age of women (34 years) whose eggs we used for our research. It is possible that female age affects the responsiveness of eggs to Sr^2+^ because eggs from older mice have a reduced ATP level compared to those from younger mice (Simsek-Duran *et al.*, 2013). Interestingly, such maternally aged eggs also undergo fewer Ca^2+^ oscillations in response to Sr^2+^ medium compared to eggs from younger mice (Haverfield *et al.*, 2016). The difference in Sr^2+^ sensitivity of mouse eggs with maternal age is not due to differences in sensitivity to PLCζ or Ca^2+^ influx and our data now suggest that it could be due to a decline in ATP levels.

It would have been useful to investigate the effect of ATP on IP3Rs in eggs by increasing cytosolic ATP levels in human eggs. However, it is not clear how this can be achieved because supplying more substrate, such as pyruvate, is not effective at increasing ATP (Dumollard *et al.*, 2009). This is not surprising because mitochondrial ATP production is controlled by feedback mechanisms on many different enzymes (Brown, 1992). In contrast to increasing ATP in human eggs, it is possible to reduce ATP levels in mouse eggs by incubation in pyruvate-free medium (Dumollard *et al.*, 2004). However, if such eggs with low ATP (presumably along with higher ADP and phosphate) undergo a transient Ca^2+^ increase they generally fail to recover to normal resting levels (Dumollard *et al.*, 2008). Hence, we could not test IP3R sensitivity directly using pyruvate-deficient mouse eggs. However, with Sr^2+^ medium we could probe the ability of the IP3R to generate Ca^2+^ release without necessarily causing any Ca^2+^ transient. When mouse eggs were ‘starved’ of pyruvate, in either of two types of medium, they consistently failed to show Ca^2+^ oscillations for 1 or 2 h in response to medium containing 5 mM or 10 mM Sr^2+^. This is unprecedented in our experience since all mouse eggs will normally undergo Ca^2+^ oscillations within minutes of placing in Sr^2+^ containing medium. Hence, the starved mouse egg’s lack of response mimics the human egg. The lack of response of mouse eggs in such medium was clearly reversed by the addition of pyruvate and this was associated with a rise in ATP levels, as reported previously (Dumollard *et al.*, 2004, 2008). This suggests that ATP plays a causal role in IP3R sensitivity. The higher level of ATP provides a simple explanation for the return of Ca^2+^ oscillations since ATP (probably as ATP^4−^) is known to promote Ca^2+^ release via the ITPR1, which is the predominant IP3R subtype found in mammalian eggs (Foskett *et al.*, 2007). It is possible that ATP^4−^ produced by mitochondria has a localized action on the ITPR1 because the membranes of mitochondria and the endoplasmic reticulum in cells can be located *c.* 20–40 nm apart (Giacomello and Pellegrini, 2016).

Parthenogenetic activation of mouse eggs routinely involves using Sr^2+^ medium, which is more effective than Ca^2+^ ionophores (Bos-Mikich *et al.*, 1995; Ferrer-Buitrago *et al.*, 2018a). We previously used a mouse model of failed ICSI to show that Sr^2+^ medium was as effective as recombinant PLCζ in activating development to the blastocyst stage (Sanusi *et al.*, 2015). Sr^2+^ medium is the most effective means of activating mouse eggs and it causes the highest rates of artificially induced pre-implantation development (Ferrer-Buitrago *et al.*, 2018a). Sr^2+^ induced mouse egg activation is simple, cost effective and easily modulated by changing concentrations and incubation times. Our work suggests that Sr^2+^ medium, as currently used in mouse eggs, will fail to activate human eggs because they have a lower ATP level. However, methods for promoting mitochondrial ATP production may enable human eggs to undergo Ca^2+^ oscillations and activate in response to Sr^2+^ medium.

## Supplementary data

Supplementary data are available at *Molecular Human Reproduction* online.

## Data availability

The datasets underlying this article will be shared on reasonable request to the corresponding author.

## Supplementary Material

gaaa086_Supplementary_Data
