# Supplementary material for: The role of ATP in the differential ability of Sr2+ to trigger Ca2+ oscillations in mouse and human eggs
Source: Mol Hum Reprod. 2021 Jan 5;27(1):gaaa086. doi: 10.1093/molehr/gaaa086 (PMC7846092; doi:10.1093/molehr/gaaa086)
Supplement: gaaa086_Supplementary_Data [file gaaa086_supplementary_data.pdf]

The role of ATP in the differential ability of Sr^{2+} to trigger Ca^{2+} oscillations in mouse and human eggs. Anna Storey, Khalil Elgmati, Yisu Wang, Paul Knaggs and Karl Swann.

Contents: Figure S1.

Human eggs in 10mM Sr^{2+} (Mg^{2+} free medium)

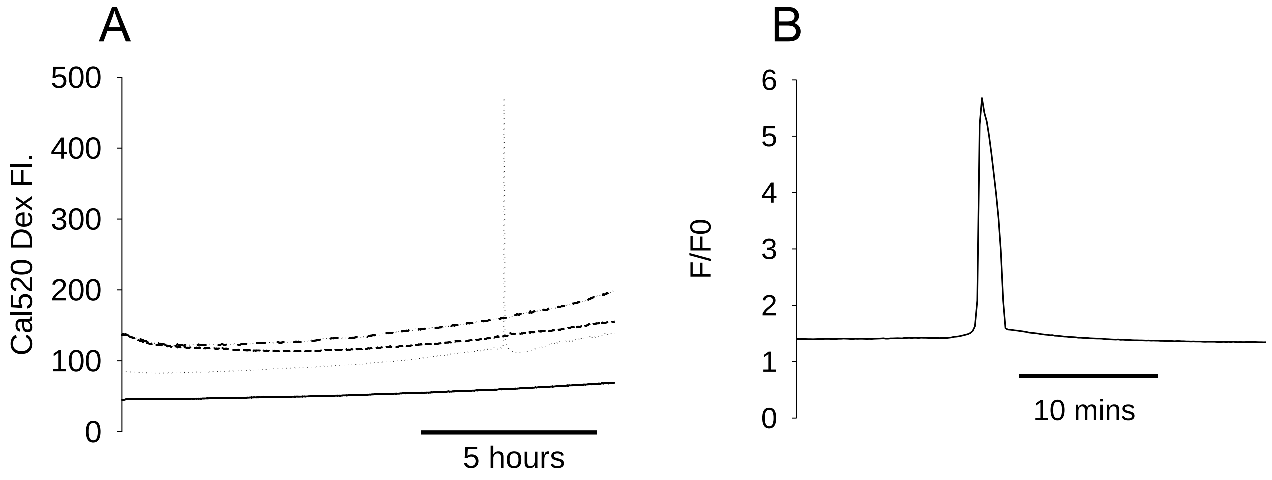

Figure S1. Ca^{2+} in human eggs in Sr^{2+} (Mg^{2+} free) medium measured with Cal520-Dex over 10 hours. Conditions are the same as those use for mouse eggs as in Fig1 F. A shows the recording from 4 human eggs in the dish (y-axis in arbitrary units) and B shows an expanded view of the one egg that showed a single Ca^{2+} spike after >10 hours.
